# Climate Change Mitigation: Assessing Strategies that Offer Potential Human Health Benefits

**DOI:** 10.1289/ehp.122-A139

**Published:** 2014-05-01

**Authors:** Julia R. Barrett

**Affiliations:** Julia R. Barrett, MS, ELS, a Madison, WI–based science writer and editor, has written for *EHP* since 1996. She is a member of the National Association of Science Writers and the Board of Editors in the Life Sciences.

Climate change mitigation strategies, including efforts to reduce greenhouse gas emissions, are not specifically designed to improve human health but could potentially do so anyway.^1^^,^^2^ A review in this issue of *EHP* critically examines different models for estimating these so-called co-benefits and highlights improvements that could help assess which mitigation strategies are the most promising for both climate and human health.^1^

Mitigation strategies to reduce greenhouse gas emissions include shifting from fossil fuels to renewable energy sources, reducing energy use and waste, and improving transportation options.^3^ These strategies may also reduce air pollution, improve water quality, and promote physical activity.^1^^,^^2^^,^^3^ Characterizing the potential scope and scale of these health co-benefits can help policy makers prioritize mitigation actions against a backdrop of finite time and resources.

**Table 1:** Examples of Mitigation Strategies and Selected Co-Benefits/Co-Harms (adapted from Remais et al.^1^)

Mitigation strategy	Potential health drivers	Potential co-benefits/co-harms
Reduce fossil fuel combustion	Reductions in conventional air pollutants	Reductions in cardiovascular morbidity/mortality, asthma/other respiratory diseases, and developmental disorders | Improved crop survival and productivity
Increase production of some types of biofuels	Increased food prices and reduced availability if biofuels compete directly with food crops	Food insecurity and malnutrition
Implement carbon capture and sequestration	Effects on groundwater availability and quality; contamination with metals/minerals and sudden carbon dioxide/hydrogen sulfide releases	Various, depending on specific contaminants
Improve fuel economy, increase adoption of electric and other noncombustion engines, and enact tighter on-road vehicle emissions standards	Reductions in conventional air pollutants	Reductions in cardiovascular morbidity/mortality and asthma/other respiratory diseases
Increase access and convenience of active modes of transportation, including walking, cycling, and public transit	Reductions in conventional air pollutants | Increased physical activity levels	Reductions in cardiovascular morbidity/mortality, asthma/other respiratory diseases, and developmental disorders | Reductions in cardiovascular morbidity/mortality, obesity, and risk of diabetes, certain cancers, dementia, depression, and injury
Reduce ruminant livestock production, and capture methane emissions	Reduction in ozone air pollution | Reduced consumption of animal products with high saturated fat, red meat, and processed meat | Increased consumption of fruits, vegetables, and unsaturated fats of vegetable origin	Reductions in cardiovascular and respiratory morbidity/mortality | Reductions in risk of certain cancers, including large bowel cancer
Increase green space and parks in built environment, and increase shading and vegetation along roads	Increased physical activity | Reduced exposure to excessive temperatures	Reductions in risk of cardiovascular events, some cancers, and mental health problems
^1^Remais JV, et al. Estimating the health effects of greenhouse gas mitigation strategies: addressing parametric, model, and valuation challenges. Environ Health Perspect 122(5):513–520 (2014); http://dx.doi.org/10.1289/ehp.1306744.		

In constructing models to estimate co-benefits, experts must consider which health factors to include and how sensitive they are to mitigation actions. They must also consider key methodological issues such as sources of uncertainty and the possibility that some mitigation actions may be accompanied by low-probability events with highly adverse health impacts (e.g., a nuclear power plant disaster). These hypotheticals are challenging to characterize and quantify.

Modelers also must interpret the results, for example, by using discount rates—complex calculations that convert anticipated future intervention costs, impacts on the climate, adverse health effects, and health cost savings, to their present-day value. “Discount rates are central to all decisions with long-term implications, and the co-benefits of mitigation activities have multiple costs and benefits distributed over time,” says lead author Justin Remais, an associate professor in environmental health at Emory University. “We need to consistently account for the relative value of near-term versus long-term benefits and costs.”

But choosing an appropriate discount rate is challenging because it is based on a number of unknowns, including future generations’ wealth. The rate must also accommodate the social values of the current generation—for instance, how much people are willing to sacrifice their own comfort for an uncertain benefit to their descendents.

Discount rates currently are not applied consistently across co-benefit models, something the authors recommend changing. They also suggest that policy makers be involved from the outset in developing models. Finally, they recommend that co-benefits modelers evaluate mitigation strategies on the basis of many criteria simultaneously, including not just health and climate impacts but also economic growth and political acceptability.

“[This review] is a logical extension of the earlier range-finding papers published in *The Lancet* several years ago,^2^ featuring the health co-benefits idea and the related public health and economics arguments,” says Anthony McMichael, professor emeritus at the National Centre for Epidemiology and Population Health, Australian National University, who was not involved in the study. “The discounting issue is particularly important and will make a great difference to the estimated longer-term cost-benefits,” he says. McMichael also highlights inclusion of water impacts and potential health co-harms as welcome additions to models, although uncertainties continue to be a problematic, yet unavoidable issue.

The need for rigorous co-benefits research and modeling is increasingly urgent because many significant mitigation policy decisions will be made worldwide in the next decade. “Policy makers need relevant, credible, and useful information regarding potential health impacts to inform these decisions,” Remais says. “Models that estimate the health co-benefits and co-harms of mitigation strategies can play a key role.”
